# Practical Uranalysis and Urinary Diagnosis

**Published:** 1895-12

**Authors:** 


phia: The F. A. Davis Co., Publishers, 1914 and 1916 Cherry Street.

Practical Uranalysis and Urinary Diagnosis: A Manual for the Use of Physicians, Surgeons, and Students. By Charles W. Purdy, M. D., Queen's University; Fellow of the Royal College of Physicians and Surgeons, Kingston; Professor of Urology and Urinary Diagnosis at the Chicago

Post-Graduate Medical School. Author of *Bright's Disease and Allied Affections of the Kidneys;* also of *Diabetes: Its Causes, Symptoms, and Treatment.* Second revised edition. With numerous illustrations, including photo-engravings and colored plates. In one crown octavo volume, 360 pages, in extra cloth, $2.50 net. Philadelphia: The F. A. Davis Co., Publishers, 1914 and 1916 Cherry Street.

This book has been reviewed before. In the August number of this journal, at page 384, a full review of it was given, to which the reader is referred. That was its first issue. The volume now before us is the *second* edition. Thus there have been two editions within a year. Surely no better recommendation of it could be given than this simple announcement that a second edition has come out within a year. What was said in our previous review is equally applicable to the present volume, and we cordially recommend it to the profession.

